# Society of Biology

**Published:** 1879-05

**Authors:** O. C. Oliver

**Affiliations:** Hotel D’Orient, Paris


### ARTICLE XII.

SOCIETY OF BIOLOGY. March 1, 1879. President, M. Paul Bert.

The president communicated to the society the results of some of his experiments on the removal of one of the cerebral hemispheres. Four axolotls were shown, two white and two black, in each of which a cerebral hemisphere had been removed six months previously. These batrachians, living in the water, had not presented any circulatory trouble. The speaker had operated upon bats in a like manner, and with negative results. There were some curious points noticed on the axolotls, however. Immediately after the operation, the white axolotl became covered with black spots, the black axolotl also became very curiously mottled, and at the present time the pigment showed a strong tendency to disappear.

*Contraction in Intra-ventricular Hæmorrhages and Injections of Liquids* —M. Cassey showed that his experiments differed from those of M. Duret, communicated at a previous meeting, in many essential particulars. First, M. Duret wished to make the injection not in the ventricles, but in the sub-arachnoidean space, but in two instances the canula, introduced too deeply, penetrated the cerebral substance to the depth of several millimeters, and the liquid injected violently in the interior of the cerebral substance, forced its way through the hemisphere to the ventricles. M. Cassey, on the contrary, has injected coagulable liquids directly into the ventricles, by the aid of a canula introduced through the corpus callosum, at its uncovered portion, i.e., corresponding to the median fissure of the brain. Secondly, M. Duret has injected a considerable quantity of liquid, thus determining ecchymoses of the ventricular surfaces. M. Cassey has never injected a quantity of liquid exceeding 10 to 20 grams, and has produced no lesion of the ventricular surfaces. Yet the animal has presented an intense and generalized contraction. Thirdly, M. Duret admits that the contraction is due to the excitation, by the injected liquid, of the flow of the fourth ventricle, and especially the corpora restiformia. M. Cassey rejects this theory, in explaining the contraction produced in his experiments, for in one very clearly marked case, the injected liquid coagulated in the middle and lateral ventricles, without having penetrated the fourth ventricle at all. M. Cassey thinks the contraction due to excitation of the subjacent excito-motor portions of the encephalon (internal capsule and cerebral peduncles) due to rapid compression by the walls of the middle and lateral ventricles. In like manner M. Cassey would explain the contraction in hæmorrhages limited to the lateral ventricles, not admitting the theory of M. Duret, which attributes the contraction in these cases to shock of the sub-arachnoid fluid, upon the floor of the fourth ventricle. In cases where the effused blood flowed into the fourth ventricle, the convulsions were no more frequent than in cases where the effusion was limited to the lateral ventricles.

In 20 cases of hæmorrhages into the lateral ventricles, in which the blood had flowed into the fourth ventricle, convulsive phenomena were observed in 8, while the 12 remaining cases showed none. O. C. OLIVER.

HOTEL D'ORIENT, PARIS, March 10, 1879.

